# HIV screening among TB patients and level of antiretroviral therapy and co-trimoxazole preventive therapy for TB/HIV patients in Hawassa University Referral Hospital: a five year retrospective study

**DOI:** 10.11604/pamj.2017.28.75.11977

**Published:** 2017-09-26

**Authors:** Asnake Simieneh, Mengistu Hailemariam, Anteneh Amsalu

**Affiliations:** 1 Department of Medical Laboratory Sciences, College of Medicine and Health Sciences, Hawassa University, Ethiopia; 2 Department of Medical Microbiology, University of Gondar, Ethiopia

**Keywords:** Tuberculosis, TB/HIV co-infection, ART, CPT

## Abstract

**Introduction:**

Initiation of antiretroviral therapy (ART) and co-trimoxazole preventive therapy (CPT) is recommended for tuberculosis (TB)/human immunodeficiency virus (HIV) co-infected patients to prevent opportunistic infection. The aim of this study was to assess the prevalence of HIV among TB patients and initiation of ART and provision of CPT for TB/HIV co-infected patients in Hawassa university referral hospital.

**Methods:**

A five year document review was done on 1961 TB patients who are registered at TB clinic of Hawassa university referral hospital from September 2009 to august 2014. Data were collected using checklist. Data analysis was done by using SPSS version 20 software. Bivariate and multivariate logistic regression analysis was used to determine the predictors of TB/HIV co-infection.

**Results:**

Among 1961 TB patients diagnosed in the hospital, 95% (1765) were screened for HIV. Of these, 13.9% (246) were HIV positive. Out of 246 TB/HIV co-infected patients 31.7% (78/246) and 37.4% (92/246) were enrolled to start ART and CPT respectively. Roughly the trends of TB/HIV co-infection decreased with increased linkage to CPT, while linkage to ART was not regular across the year. The rate of TB/HIV co-infection was significantly associated with type of TB.

**Conclusion:**

Although, trend of HIV among TB patients has decreased across the year, only a minority of co-infected patients was linked to start ART and CPT. Therefore, screening of all TB patients for HIV and linkage of co-infected patients to HIV care to start ART and CPT should be strengthened in-line with the national guidelines.

## Introduction

Tuberculosis (TB) ranks alongside human immunodeficiency virus (HIV) as a leading cause of death worldwide. It causes ill-health among millions of people each year. Globally, 9.6 million new TB cases were estimated, in 2015. Of these, 28% were from the African region. In the same year 12% of the total new TB cases were HIV-positive. The African region accounted for 74% of the estimated number of HIV-positive incident TB cases [1]. The interaction between TB and HIV infection is complex; HIV infection weakens the immune system and increases the susceptibility to TB. The presence of HIV enhances the reactivation and progression of latent *Mycobacterium tuberculosis* to overt TB disease, and having TB disease accelerates HIV disease progression [2]. The first key intervention for reducing the burden of HIV-associated TB is HIV testing for TB patients. Globally, 51% of notified TB patients had a documented HIV test result in 2014, of these 16% were HIV positive [1]. The prevalence of HIV co-infection among TB patients is highest in the African region, where it continues to increase and reached 79% in 2014, up from 78% in 2013 [1]. In Ethiopia, 10-67% of TB patients with an HIV test result were HIV positive [3–6]. Collaborative TB/HIV activities are essential to reduce the burden of HIV among TB patients [7]. The first priority as soon as HIV is identified in a TB patient is to initiate TB treatment, followed by CPT as soon as possible and ART within the first two to eight weeks of treatment, regardless of the CD4 count [8]. According to the WHO report in 2014, about 77% and 87% of HIV positive TB patients were enrolled on CPT and ART, respectively [1]. Despite, the implementation of TB/HIV collaborative activities in Ethiopia [9], few studies have reported prevalence of HIV among TB patients and level of HIV positive TB patients enrolled on CPT [4], to our knowledge, there are no reports in the study area. Therefore this study was conducted to determine the prevalence of HIV among TB patients and level of HIV positive TB patients enrolled on CPT and ART in Hawassa university referral hospital, southern Ethiopia.

## Methods

### Study design, area and period

A five year document review was conducted on 1961 tuberculosis patients diagnosed at Hawassa University referral Hospital from September 2009 to August 2014. The hospital is located in Hawassa city 273 km south of Addis Ababa. This hospital serves the population of Hawassa and extends to include surrounding areas encompassing roughly 17 million people.

### Study population

The study population included all TB cases registered from September 2009 to August 2014 at Hawassa University referral hospital DOTs clinic. Patients were diagnosed, registered, treated and referred to other DOTs clinics following the national tuberculosis, leprosy and TB/HIV prevention and control program guideline [8].

### Data collection

Data on socio-demography (age and sex), type of tuberculosis infection, patient’s category, treatment follow-up center, baseline ART and CPT provision for HIV-infected TB patients and were collected from April to May 2015 using a checklist prepared for this purpose. Patients with incomplete information were excluded.

### Data analysis

The data was coded, entered and analyzed by using IBM SPSS version 20 (IBM, USA). We used frequency to compare patient characteristics and logistic regression model was used to determine the predictors of TB/HIV co-infection. Linear trends for TB/HIV co-infection, initiation of ART, provision of CPT and frequency TB patients across the year were explored using chi-square tests. Statistical significance for all analyses was set at the p = 0.05 level and 95% confidence intervals (CI) were calculated throughout.

### HIV testing and status

HIV testing in Hawassa university referral hospital was performed following the national HIV test algorithm in Ethiopia, where KHB (Shangai Kehua Bio-engineering Co, Ltd. China) was used for the first screening and positive samples were re-tested with STAT pack (Chembio HIV1/2 STAT pack Assay, USA). Samples giving discordant results in the two tests (KHB and STAT pack) were retested using tie-breaker (Unigold).

### Ethical consideration

The study was approved by the Department of Medical Laboratory Sciences ethics committee, College of Medicine and Health Sciences, Hawassa University. After obtaining permission and waiver from the hospital administration, we started data collection from DOTS clinic. Patient records/information was anonymized and de-identified prior to analysis. All information obtained from patient’s record was kept confidential.

**Abbreviations**: ART: Antiretroviral treatment; CPT: Co-trimoxazole prophylaxis treatment; DOTS: Directly observed therapy short-course; EPTB: Extra-pulmonary tuberculosis; HIV: Human immunodeficiency virus; SNPTB: Smear-negative pulmonary tuberculosis; SPPTB: Smear-positive pulmonary tuberculosis; TB: Tuberculosis

## Results

### Characteristics of patients

A total of 1961 TB patients data was reviewed. Of these, 1157 (59%) were male, with male to female ratio 1.4:1.Their mean age was 27.3 (standard deviation (SD),) years, range (0–100 years), and majority of the study participants 1297(66.1%) were aged from 15-39 years old. Among the total TB patients, 49.3% (966) had extra pulmonary TB followed by 33.3% (653) smear negative pulmonary TB patients and 16.8% (329) smear positive pulmonary while, only 0.7% (13) were unknown. The majority of TB patients were new 1572(80.2%) and only 273(13.9%) had followed their treatment in the hospital TB clinic while the rest 1688(86.1%) were transferred out to other health institution (Table 1).

**Table 1:** Characteristics of the study participant registered at TB clinic, Hawassa university referral hospital from September 2009 - August 2014

Characteristics		Frequency	Percent
Sex	Male	1157	59.0
	Female	804	41.0
Age	0-14	285	14.5
	15-39	1297	66.1
	>40	379	19.3
TB type	SPP TB	329	16.8
	SNPTB	653	33.3
	EP TB	966	49.3
	Unknown	13	0.7
Category of TB	New	1572	80.2
	Relapse	42	2.1
	Treatment failure	3	0.2
	Unknown	344	17.5
Rx follow up	DOTs at TB clinic	273	13.9
	Transferred out	1688	86.1

EPTB: Extra-pulmonary tuberculosis; HIV: Human immunodeficiency virus; SNPTB: Smear-negative pulmonary tuberculosis; SPPTB: Smear-positive pulmonary tuberculosis;DOTS= directly observed therapy short-course.

### HIV screening at TB clinic

Out of the total 1961TB patients 94.7% (1858/1961) had offered HIV test by healthcare providers. Of whom 95.0% (1765/1858) were tested for HIV and 13.9% (246/1765) were positive for HIV. While 93 (4.8%) were refused the test and 10(0.5%) have unknown HIV test result (Table 2). Among HIV positive TB patients, 37.4% (92) were linked for CPT provision. Regarding ART initiation, 17.9% (44/246) were known to have started and 13.8% (34) had already started ART before diagnosed for TB disease; hence a total of 78(31.7%) TB patients were on ART treatment and the rest 68.3% (168) had no known records of ART initiation (Table 2).

**Table 2:** HIV testing and status among TB patients at Hawassa University referral hospital from September 2009 – August 2014

Variables	Number (%)
**Total TB patients registered**	
HIV test offered	1858(94.7)
HIV test refused	93(4.8)
Unknown	10(0.5)
**HIV test result**	
Reactive	246(13.9)
Non-reactive	1519(86.1)
**CPT provision**	
Yes	92(37.4)
No mention	154(62.6)
**ART initiation**	
Started	44(17.9)
Continued	34(13.8)
Not started	168(68.3)

TB=tuberculosis, CPT= co-trimoxazole preventive therapy, ART= antiretroviral therapy

### Trends of HIV, initiation of ART and CPT for co-infected patients

**Figure 1:**
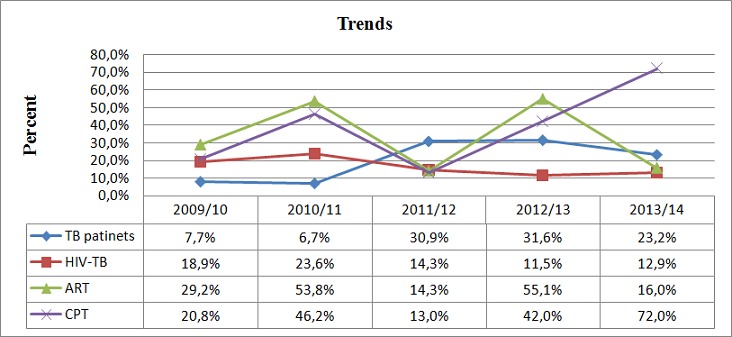
Trends of HIV among TB patients and initiation of ART and CPT for TB/HIV patients at Hawassa University referral hospital from September 2009 – August 2014

In this study, high proportion of TB patients were diagnosed in September 2011 to August 2012, 606(30.9%) and in September 2012 to August 2013, 619(31.6%). Significantly declining trends of TB/HIV co-infection (p=0.006) and increasing trends of CPT provision (p = 0.0001) among co-infected patients were observed over the five years of study period. The seroprevalence of HIV was 18.9% in 2009/10 and increased to 23.6% in 2010/11 however, subsequently decreased to 14.3% in 2011/12 to 11.5% in 2012/13 and slightly increased 12.9% in 2013/14. Provision of CPT for TB/HIV co-infected patients was increased from 20.8% in 2009/10 to 46.2% in 2010/11 and further increased to 72% in 2013/14 except in the year 2011/12 in which significantly decreased to 13%. The initiation of ART for TB/HIV co-infected patients was not regular across the year (Figure 1).

### Predictors of TB/HIV co-infection

In this study as shown in Table 3, relative to extra pulmonary TB patients, the odds of TB/HIV co-infection were about 1.5 times higher among smear-negative pulmonary TB (SNPTB) patients (aOR = 1.51; 95% CI 1.107-2.066; p = 0.009) and 2 times higher among smear-positive pulmonary TB(SPPTB) patients (aOR =1.94; 95% CI 1.348-2.790; p = 0.001). Female TB patients were more likely HIV infected than male TB patients though the difference was marginally non–significant (aOR =1.29; 95% CI 0. 983-1.702; p = 0.067) and preponderance of TB/HIV co-infection occurs with increasing of age, but the difference was not statistically significant.

**Table 3:** Predictors of TB/HIV co-infection (n=1765) among TB patients at Hawassa university referral hospital from September 2009 - August 2014

Variables	TB/HIV co- infection		COR (95%CI)	P-value	AOR (95% CI)	P-value
	No (%)	Yes (%)				
**Sex**						
Male	894(87.1)	132(12.9)	1		1	
Female	625(84.6)	114(15.4)	1.24(0.942-1.619)	0.126	1.29(0. 983-1.702)	0.067
**Age(years)**						
0-14	224(89.6)	26(10.4)	1		1	
15-39	1002(85.6)	168(14.4)	1.44(0.932-2.238)	0.100	1.37(0.882-2.140)	0.160
≥40	293(84.9)	52(15.1)	1.53(0.926-2.526)	0.097	1.40(0. 842-2.320)	0.195
**Type of TB**						
EPTB	778(88.9)	97(11.1)	1		1	
SPPTB	229(80.4)	56(19.6)	1.96(1.368-2.813)	0.001	1.94(1.348-2.790)	0.001
SNPTB	501(84.6)	91(15.4)	1.46(1.071-1.981)	0.016	1.51(1.107-2.066)	0.009
Unknown	11(84.6)	2(15.4)	1.46(0.319-6.677)	0.627	1.45(0.315-6.665)	0.635
**TB category**						
New	1228(86.5)	191(13.5)	1			
Relapse	33(86.8)	5(13.2)	0.971(0.374-2.517)	0.957		
Failure	2(66.7)	1(33.1)	3.203(0.289-35.49)	0.341		
Unknown	256(83.9)	49(16.1)	1.221(0.868-1.718)	0.234		

TB-tuberculosis; EPTB–extra pulmonary; SPPTB–smear positive pulmonary TB; SNPTB-smear negative pulmonary TB; COR –crude odds ratio; AOR-adjusted odds ratio;

## Discussion

HIV testing for all TB patients followed by provision of CPT and early initiation of ART for those found to be HIV positive has dramatically reduce mortality [6, 8]. In this study, 95% of TB patients knew their HIV status. Of these, 13.9% were positive for HIV. Out of the co-infected patients, 37.4% and 31.7% were linked to start CPT and ART respectively at the time of anti-TB treatment follow-up. Being SNPTB and SPPTB patient are predictors of TB/HIV co-infection. Significantly declining trends of TB/HIV co-infection and increasing trends of CPT linkage were observed among co-infected patients. In this study, 95% of TB patients were screened for HIV in agreement with a study in Arsi Negele Health center (94.7%) [3] and Oromia region (98%) [10]. This result is higher than the WHO report for Ethiopia (71%) [11], a national TB/HIV sentinel surveillance report from 2011-2012(86%) [8] and a study in Addis Ababa (87.1%) [4]. However, WHO were planning to screen 100% of the TB patients for HIV by 2015. On the other hand, 4.6% of TB patients refused the test in this hospital, which deserves concern. The sero-prevalence of HIV in the current study was in agreement with the previous study conducted at Arsi Negele Health center (10%) [3] and Enfrazh Health center (11.7%) [6] but lower as compared to several previous studies in Ethiopia in which the sero-prevalence of HIV positivity among TB patients ranges from 18.5% to 67% [5,12] and in other African countries such as: 42% in Uganda [13], 47.2% in Ghana [14], 61% in Kenya [15] and 56% in Malawi [16]. These differences may be attributed to differences in the prevalence of HIV infection at the community level [17]. This study also demonstrated that TB/HIV co-infection rate was significantly decreased across the years consistent with the declining trend of national HIV infection in the general population from 1.5% in 2011 to 1.1% in 2015(2014 spectrum projection) in Ethiopia [18]. To prevent opportunistic infections, the WHO recommends CPT prophylaxis for HIV-infected individuals [19]. CPT is a combination of two antimicrobial drugs that is active against a range of bacterial, fungal, and parasitic infections; prophylactic antimicrobials are taken to prevent infection [19]. In this study, 37.4% of the HIV positive TB patients have been linked for CPT. This finding is lower than previous studies in a referral hospital in North-West Ethiopia (45.9%) [20], Addis Ababa (81%) [21] and health facilities in Addis Ababa (54.3%) [4]. Recent WHO report also revealed high levels of enrolment on CPT [1]. This might be due to large number of transfer out cases and poor feedback system.

Despite the well-known benefit of ART and the integrated TB/HIV care in our hospital, only 31.7% of TB/HIV co infected patients were enrolled for ART treatment. This finding is lower than a study in Malawi(38%) [22]. The low ART enrollment is likely due to the patients might be reluctant to take ART and TB drugs simultaneously due to concerns about pill burden and drug interactions. The occurrence of TB/HIV co-infection was almost 2 times higher in SPPTB cases and 1.5 times higher in SNPTB cases than EPTB cases. This finding was in contrast with studies conducted elsewhere in Ethiopia [17,23] which showed that high prevalence of co infection was observed in patients with extra-pulmonary cases. This difference might be due to the proportion of SPPTB cases were small as compared to EPTB cases in our study. Moreover, high percentage of SPPTB cases refused HIV test as compared to EPTB cases which might decrease the prevalence of HIV among SPPTB cases (data not shown). The preponderance of TB/HIV co infection among women compared to men in our study was concordant with findings reported elsewhere [24]. In contrast, similar rate of infection by gender was also shown in Ethiopia [23,25]. In Ethiopian context where HIV is contracted primarily by heterosexual exposure and women are disproportionally infected. This study has some limitations which need to be noted while interpreting the findings. As this study was based on a retrospective review of TB registers and only 13.9% of the TB patients initiate and continue TB treatment at Hawassa university referral hospital TB clinic; the rest diagnosed at this hospital but choose to continue their treatment at the peripheral health facilities around Hawassa, we didn’t assess the effect of ART and CPT provision on treatment outcome of co-infected patients.

## Conclusion

Despite, integrated TB/HIV setting, expanded ART coverage and decreased trend of TB/HIV co-infection, only a minority of co-infected patients were linked to start ART and CPT. Therefore, screening of all TB patients for HIV and linkage of co-infected patients to HIV care to start ART and CPT should be strengthened in-line with the national guidelines.

### What is known about this topic

- The first key intervention for reducing the burden of HIV-associated TB is HIV testing for TB patients. Globally, 51% of notified TB patients had a documented HIV test result in 2014, of these 16% were HIV positive;
- The prevalence of HIV co-infection among TB patients is highest in the African region; about 79% in 2014 and in Ethiopia it is estimated 10–67%;
- According to the WHO report 2014, about 77% and 87% of HIV positive TB patients were enrolled on CPT and ART, respectively.

### What this study adds

- In this study, 95% of TB patients knew their HIV status. Of these, 13.9% were positive for HIV. Out of the co-infected patients, 37.4% and 31.7% were linked to start CPT and ART respectively at the time of anti-TB treatment follow-up;
- Despite the well-known benefit of ART and the integrated TB/HIV care in our hospital, only 31.7% of TB/HIV co infected patients were enrolled for ART treatment.

## Competing interests

The authors declare no competing interest.
